# Food intake patterns and cardiovascular risk factors in Japanese adults: analyses from the 2012 National Health and nutrition survey, Japan

**DOI:** 10.1186/s12937-017-0284-z

**Published:** 2017-09-19

**Authors:** Nay Chi Htun, Hitomi Suga, Shino Imai, Wakana Shimizu, Hidemi Takimoto

**Affiliations:** Department of Nutritional Epidemiology and Shokuiku, National Institutes of Biomedical Innovation, Health and Nutrition, 1-23-1 Toyama, Shinjuku-ku, Tokyo, 162-8636 Japan

**Keywords:** Food intake pattern, Cardiovascular risk factors, Blood pressure, Blood lipid profiles, Japanese, National Health and nutrition survey (NHNS)

## Abstract

**Background:**

There is an increasing global interest in the role of Japanese diet as a possible explanation for the nation’s healthy diet, which contributes to the world’s highest life-expectancy enjoyed in Japan. However, nationwide studies on current food intake status among general Japanese population have not been established yet. This study examined the association between food intake patterns and cardiovascular risk factors (CVRF) such as waist circumference (WC), body mass index (BMI), blood pressure (SBP, DBP), HbA1c and blood lipid profiles among general Japanese adults.

**Methods:**

De-identified data on the Japan National Health and Nutrition Survey (NHNS) 2012 with a total of 11,365 subjects aged 20–84 years were applied. Food intake patterns were derived by principal component analysis (PCA) based on 98 food groups. Generalized linear regression analysis was used to assess the relation between the food intake patterns and CVRF.

**Results:**

We identified three food intake patterns: traditional Japanese, Westernized, and meat and fat patterns. Traditional Japanese pattern was significantly related to high WC and BMI in men, and high DBP in women. Westernized pattern was associated with lower SBP, but high total cholesterol and LDL cholesterol in both men and women. Meat and fat pattern was associated with high WC, high BMI, high blood pressure and blood lipid profiles in both men and women (trend *P* < 0.001).

**Conclusion:**

The significant association between cardiovascular disease risks and three food intake patterns derived from the NHNS, showed a similar tendency to other dietary survey methods.

## Background

Cardiovascular disease (CVD) is one of the major contributors to the global burden of disease, and is the leading cause of premature mortality [1]. The vast majority of cardiovascular disease is explained by conventional risk factors such as cigarette smoking, hypertension, obesity, diabetes, dyslipidemia, and unhealthy lifestyle behaviors including poor dietary habits, excessive caloric intake, physical inactivity, and psychosocial stressors [2, 3]. Along with lifestyle factors such as physical activity, diet is one of the most important modifiable risk factors [4–7], providing effective means to achieve healthy and nutritious diets are essential for cardiovascular disease prevention [8].

Japan is unique among developed countries because the prevalence of cardiovascular disease morbidity and mortality remain substantially lower despite having the same trend in cardiovascular risk factors (CVRF) (such as rise in serum cholesterol, etc.) as other developed countries [9, 10]. The world’s highest healthy life expectancy enjoyed in Japan may be partly explained by the Japanese diet. For example, a very recent cohort study found that the food intake patterns of Japanese adults who followed the government recommended food guide for the nation (the Japanese Spinning Top Food Guide 2005) at the baseline, had lower rates of mortality than those who didn’t [11]. And, another nationwide study in elderly Japanese has observed that improvement in dietary habits, such as yearly increase in vegetables and meat intake, may contribute to decreasing prevalence of anemia [12].

As dietary habits are influenced by lifestyle, socioeconomic and environmental factors such as family income, food prices, individual preferences, cultural beliefs, traditional single food- or nutrient-based approach may fail to take into consideration the complicated interaction and cumulative effects among nutrients. Therefore, overall assessment of food intake patterns, taking into account of the interactions, inter-correlations between nutrients and foods and their cumulative effects, has gained attention in the studies of the association between health and diseases [13].

Many epidemiologic cohort studies have been implemented to explain the correlation between food intake patterns and cardiovascular risk factors such as hypertension, obesity and blood lipid profiles [14, 15]. The scientific evidences have consistently shown that the food intake pattern rich in fruits, vegetables, fish and whole grains (known as healthy or prudent pattern) [16–18], DASH diet (Dietary Approaches to Stop Hypertension) [19] and Mediterranean diet [20–23] are favorable for reducing cardiovascular disease risks, whereas food intake patterns characterized by high fat and sugar or a meat-based diet have deleterious effects and have been associated with increased risks of obesity, type 2 diabetes, and cardiovascular disease [15, 24]. To date, only one cross-sectional study in Japanese reported that vegetable-rich diet pattern was significantly associated with favorable blood lipid profiles in women [25]. Most of the above mentioned cohort studies identified food intake pattern using intake data from semi-quantified food frequency questionnaires (FFQs), on limited populations. Because FFQs are developed to assess the habitual intakes of the specific population of research subjects, the food lists (5 to 350, median number 79) applied may not be able to reflect the intakes of the general population [26]. On the other hand, weighed dietary records are able to provide more details on actual intakes and provide more precise estimates of portion sizes than FFQs. The Japan National Health and Nutrition Survey (J-NHNS) is unique, as it is based on a single day dietary survey in which the survey participants are requested to weigh and record all foods consumed. Currently 2116 food items are available for use in the J-NHNS. Of these, 1789 food items are the same as those listed in the Standard Tables of Food Composition 2010 [27], and 50 food items are original for the survey. The foods are then grouped into 98 food groups, which has been consistently used since 2005 [28].

This study aimed to explore the food intake patterns and examine their association with cardiovascular risk factors such as waist circumference, body mass index (BMI), blood pressure, HbA1c level and blood lipid profiles among 11,365 Japanese aged 20–84 years, using the nationally representative data from J-NHNS conducted in year 2012, while controlling for a wide range of potential confounding factors.

## Methods

### Survey outlines of the 2012 J-NHNS

Out of the 2010 national census areas units, 475 areas stratified by prefecture were randomly extracted for the 2012 J-NHNS, consisting of 10 areas per prefecture (Tokyo: 15 areas). Approximately 50 households were included in each area. Participants were household members (aged 1 year and over) of all households residing in the selected area. Total number of households and family members aged 1 year and over in the 475 areas were approximately 23,750 and 61,000, respectively. Of the selected census areas, four alternative areas were re-selected to replace those unable to conduct this survey due to the influence of the Great East Japan Earthquake [28].

De-identified records on the 2012 J-NHNS with the permission of secondary use of data from Ministry of Health, Labor and Welfare were applied. A total of 11,365 subjects, 4686 men and 6679 women (pregnant and lactating mothers were excluded) aged 20–84 years who had a complete data on dietary intake, lifestyle factors, anthropological and blood pressure measurements, HbA1c measured in National Glyco-hemoglobin Standardization Program (NGSP) units (%), fasting blood lipid profiles [total cholesterol (TC), low-density lipoprotein cholesterol (LDL-C), high-density lipoprotein cholesterol (HDL-C)] were selected.

The dietary survey was conducted by semi-weighing of all foods consumed in the one-day dietary records of the household, with proportional distribution within the household members [29]. Information regarding the cooking status (boiled, roasted, etc.) of each food was recorded, and post-cooked nutrient values in the Standard Food Composition Tables 2010 were applied for estimating nutrient intakes. For foods categorized as “cereals” except bread, post-cooked weight of the food was applied as the intake food weight. Participants aged 20 years and above were asked to record the number of daily step counts measured with a pedometer as part of the physical examination. BMI was calculated by weight in kilograms divided by height in meters squared.

### Definition of hypertension, diabetes, hypercholesterolemia and elevated LDL cholesterol

Hypertension was defined as either having a systolic blood pressure (SBP) of ≥140 mmHg or diastolic blood pressure (DBP) of ≥90 mmHg, currently under anti-hypertensive treatment, or previously diagnosed for hypertension. We defined diabetes based on HbA1c ≥6.5% or currently under anti-diabetic treatment, or previously diagnosed for diabetes. Subjects with hypercholesterolemia were defined as having serum total cholesterol level ≥ 240 mg/dL and subjects with elevated LDL cholesterol were defined as having serum LDL cholesterol level ≥ 140 mg/dL or subjects having lipid lowering medication for both. In the present study, anti-hyperlipidemia medication included both cholesterol-lowering medication and triglyceride-lowering medication.

### Statistical analysis

The original 2116 food items used in the J-NHNS are categorized into 98 food groups [28]. Then food intake patterns were identified using principal component analysis (PCA) based on the 34 food groups reorganized from the original 98 food groups (Table 1). Analyses were done after excluding the subjects with total energy intake (kcal) under the 5th percentile (*n* = 1281, 1124 kcal) or above the 95th percentile (n = 1281, 2792 kcal). Eigen values > 1.5 were used to determine whether a factor should be considered as major food intake pattern. Varimax rotation was applied to review the correlations between variables and factors. Food groups with positive loadings in each pattern indicate the direct relationship and food groups with negative loadings shows the inverse relationship with that pattern. For each subject, the factor scores for each food intake pattern were calculated by summing the intakes of food items weighted by their factor loading [13]. Factor scores were then categorized into four groups based on the quartiles of factor scores. Spearman’s correlation coefficients were calculated between the factor scores of each pattern and energy-adjusted nutrient intakes so that the correlation between food intake patterns and specific nutrient intakes could be studied.

**Table 1:** Factor loadings and explained variation in food groups

Food groups		Factor1	Factor2	Factor3
34 Food groups used in dietary pattern anaylsis	Original 98 food items from J-NHNS [food group number]	Traditional Japanese	Westernized	Meat and fat
Rice	Rice [1]	0.149	**−0.667**	−0.021
Rice products	Rice products [2]	−0.003	0.061	−0.030
Flour and wheat products	Flour and wheat [3], other wheat products [4]	−0.064	−0.038	**0.399**
Bread and danish	bread [4], danish [5]	−0.207	**0.638**	0.006
Noodles (includes soba)	*Udon*, chinese noodles [6] instant chinese noodles [7] pasta [8] *soba* (buckwheat noodles) [10]	−0.063	0.224	0.282
Corn products and other grains	Corn products [11] other grains [12]	0.022	0.058	−0.022
Potatoes	Sweet potatoes [13], potatoes [14], other starchy roots and tubers [15], processed starch [16]	**0.323**	−0.042	0.051
Sugars and sweeteners	Sugars and sweeteners [17]	**0.394**	0.203	0.098
Beans	Soy beans and soybean products [18], Tofu [19], soy bean curd [20], Natto [21], other soy bean products [22], other beans [23]	**0.381**	−0.043	−0.097
Seeds and nuts	seeds and nuts [24]	0.163	0.115	−0.035
Fresh vegetables	tomato [25] carrot [26] spanich [27] green pepper [28] other brightly colored vegetables [29] cabbage [30] cucumber [31] radish [32] onion [33] Chinese cabbage [34] other light-colored vegetables [35]	**0.577**	0.049	0.162
Vegetable juice	vegetable juice [36]	0.006	0.116	−0.010
Salted or pickled vegetables	leafy vegetables pickles [37] pickled radish and other salted or pickled vegetables [38]	0.261	−0.148	−0.177
Fresh fruit	Strawberry [39], citrus fruits [40], banana [41], apples [42], other fruits [43]	**0.389**	**0.333**	−0.288
Jam	Jam [44]	0.057	**0.356**	−0.041
Fruit juice	Fruit juice [45]	−0.055	0.036	0.050
Mushrooms	Mushrooms [46]	**0.372**	0.085	0.117
Seaweeds	Seaweeds [47]	0.262	−0.033	−0.049
Fish and shellfish	Mackerel, sardines [48], salmon and trout [49], red snapper, flounder and flatfishes [50], tuna, swordfishs [51], other raw fishes [52], shellfishes [53], squid and cuttlefish [54], prawns and crabs [55], processed fishes and seafood (salted, dried) [56], canned fish and seafoods [57] *tsukudani* [58] fish paste [59], fish ham and sausages [60]	**0.389**	−0.144	−0.189
Meat	Beef [61], pork [62], ham and sausages [63], other meats [64], chicken [65], other poultry [66], innards [67], whale [68], other processed meat and meat products [69]	−0.055	−0.114	**0.623**
Eggs	Eggs [70]	0.095	−0.114	0.238
Milk, cheese, and yogurt	Milk [71], cheese [72], and yogurt [73]	0.144	**0.420**	−0.160
Other milk products	Other dairy products [74], other milks [75]	−0.076	0.045	0.034
Butter and margarine	Butter [76], margarine [77]	−0.160	**0.484**	0.164
Other fats	Vegetable oils [78], animal fats [79], other fats [80]	−0.032	−0.162	**0.573**
Confectionaries	Japanese confectioneries (*Wagashi*) [81], cakes and pastries [82], biscuits [83], candies [84], other confectioneries [85]	−0.019	0.203	−0.139
Alcohol drinks	Japanese *sake* [86], beer [87], whiskey, wine and other alcoholic beverages [88]	0.028	−0.169	0.247
Tea	Tea [89]	0.269	−0.003	−0.232
Coffee and cocoa	Coffee and cocoa [90]	−0.052	0.282	0.141
Other beverages	Other beverages [91]	−0.091	−0.018	0.192
Sauce, mayonnaise	Sauce [92], salt [94], mayonnaise [95], other seasonings [97]	0.161	0.146	**0.389**
Soy sauce	Soy sauce [93]	**0.554**	−0.014	0.242
Miso	Miso [96]	**0.397**	**−0.309**	−0.139
Spices	Spices [98]	0.137	0.019	0.149
**Explained variation in food groups (%)**		2.080	2.007	1.729

Factor loadings (absolute values) > 0.3 were shown in bold characters

A detailed description of food groups used in NHNS were shown on page 57–63 (Table 1) of reference [28]

Generalized linear models was used to assess the association of adherence to three major food intake patterns with waist circumference, body mass index (BMI), systolic and diastolic blood pressure, HbA1c and blood lipid profiles (TC, LDL-C and HDL-C) after adjustment with other potential confounding variables; such as age in years, BMI, step counts/day (as continuous variables), smoking habit (1: current smoker, 2: past smoker, 3: non smoker), drinking habit (1: Yes, 2: No), and, medication status (hypertension, diabetes, and dyslipidemia, 1:Yes, 2: No). Trend association across quartile categories of each food intake pattern was assessed by using Cochrane-Armitage trend test for categorical variables and generalized linear regression analysis for continuous variables. Logistic regression analysis was used to determine the association of dietary patterns with the risk of hypertension, hypercholesterolemia and elevated LDL cholesterol. The odds ratios (OR) were estimated for each quartile compared with the lowest quartile of each food intake pattern as the reference. The association analyses were performed separately in men and women. Two-sided *P* values <0.05 were regarded as statistically significant. All statistical analyses were performed using Statistical Analysis System 9.3 (SAS Institute, Cary, NC, USA).

## Results

### Food intake pattern analysis

We identified three food intake patterns by principal component analysis: (a) “traditional Japanese” (greater intake of miso, soy sauce, fresh vegetables and fruits, beans and potatoes), (b) “Westernized” (greater intake of bread, dairy, butter and margarine, jam, low intake of rice and miso), (c) “Meat and fat” (high intake of meat, fat, sauce and mayonnaise, wheat and wheat products). These three food intake patterns accounted for 2.1%, 2.0% and 1.7%, respectively, explained 5.8% of the total variance in food intake (Table 1).

Spearman’s correlation coefficients showed positive correlations between nutrients and traditional Japanese pattern, while Westernized pattern showed positive correlation with total fat intake, micronutrients except vitamin D, vitamin B_12_, sodium and iron, and negative correlation with carbohydrate, ω-3 fatty acid. Meat and fat patterns showed positive correlation with total energy, protein and fat intake, ω-6 fatty acids, vitamin B_1,_ sodium and iron, and negative correlation with other micronutrients (Table 2).

**Table 2:** Correlation coefficients ^a^between subject characteristics, dietary energy and nutrient intakes and each factor

	Men			Women		
	Traditional Japanese	Westernized	Meat and fat	Traditional Japanese	Westernized	Meat and fat
Age at survey (yrs)	0.321	0.108	−0.378	0.354	0.024	−0.316
Height (cm)	−0.133	−0.011^b^	0.253	−0.162	0.063	0.209
Body weight (kg)	−0.007^b^	−0.019^b^	0.152	−0.008^b^	−0.004^b^	0.080
Abdominal circumference (cm)	0.095	0.003^b^	−0.025	0.127	−0.032	−0.078
BMI	0.071	−0.020^b^	0.016^b^	0.086	−0.047	−0.039
Steps per day	0.022^b^	−0.008^b^	0.125	0.003^b^	0.063	0.123
Systolic blood pressure (mmHg)	0.112	−0.028	−0.091	0.207	−0.038	−0.135
Diastolic blood pressure (mmHg)	−0.017^b^	−0.042	0.056	0.107	−0.001^b^	−0.027
Serum total cholesterol (mg/dL)	−0.027	0.023^b^	0.129	0.094	0.123	−0.009^b^
Serum HDLcholesterol (mg/dL)	−0.003^b^	−0.004^b^	0.092	−0.048	0.100	0.093
Serum LDL cholesterol (mg/dL)	−0.063	0.051	0.094	0.056	0.093	−0.011^b^
HbA1c (%)	0.156	0.049	−0.142	0.193	0.009^b^	−0.120
Energy and nutrient intakes						
Energy (kcal)	0.357	−0.065	0.313	0.405	0.102	0.234
Protein (g)	0.534	0.011	0.212	0.534	0.076	0.156
Fat (%energy)	0.090	0.138	0.469	0.105	0.209	0.429
ω-3 fatty acids (g)	0.338	−0.117	0.008^b^	0.348	−0.091	0.008^b^
ω-6 fatty acids (g)	0.153	0.016^b^	0.448	0.194	0.046	0.397
Carbohydrates (%energy)	0.331	−0.083	0.008^b^	0.416	0.037	−0.047
Dietary fiber (g)	0.680	0.226	−0.008^b^	0.715	0.239	−0.013^b^
Vitamin A (μgRAE)	0.393	0.141	0.026	0.417	0.149	0.015^b^
Vitamin D (μg)	0.392	−0.072	−0.207	0.374	−0.053	−0.168
Vitamin B_1_ (mg)	0.322	0.150	0.239	0.393	0.182	0.213
VitaminB_2_ (mg)	0.449	0.165	−0.002^b^	0.475	0.195	−0.043
Vitamin B_6_ (mg)	0.600	0.030	0.068	0.631	0.088	0.024
Vitamin B_12_ (μg)	0.353	−0.074	−0.157	0.351	−0.051	−0.149
Folate (μg)	0.678	0.097	−0.075	0.708	0.119	−0.105
VitaminC (mg)	0.522	0.174	−0.154	0.552	0.180	−0.176
Sodium (mg)	0.519	0.025	0.184	0.555	0.029	0.152
Potassium (mg)	0.751	0.205	−0.020^b^	0.768	0.240	−0.057
Calcium (mg)	0.514	0.307	−0.107	0.531	0.322	−0.120
Iron (mg)	0.692	0.018^b^	0.038	0.708	0.044	0.009^b^

^a^Spearman’s correrlation analysis

^b^not significant

### General characteristics of the subjects by food intake patterns

Table 3 shows the general characteristics of the study subjects according to quartile categories of each food intake pattern score. Subjects with a higher score for the traditional Japanese pattern were older, had higher BMI and waist circumference, higher SBP, lower LDL-C, higher proportion of having anti-hypertensive, anti-diabetic, anti-lipid medication and were likely to be past smoker in men and less likely to have smoking and drinking habits in women. Subjects with a higher score for the Westernized pattern were older, had lower BMI and waist circumference, higher LDL-C, were likely to have anti-hypertensive and anti-lipid medications, and were likely to have drinking habit than the group with lower Westernized pattern score. Participants with higher meat and fat pattern score were younger, had higher step counts, less likely to take anti-hypertensive, anti-diabetic and anti-lipid medications, but more likely to have smoking and drinking habits.

**Table 3:** Subject charcterisics by gender, according to quartiles of the three food intake pattern scores

	Traditional Japanese									Westernized									Meat and fat								
	***Q1***		***Q2***		***Q3***		***Q4***			***Q1***		***Q2***		***Q3***		***Q4***			***Q1***		***Q2***	***Q3***			***Q4***		
	*Mean*	*S.D.*	*Mean*	*S.D.*	*Mean*	*S.D.*	*Mean*	*S.D.*	*p for trend*	*Mean*	*S.D.*	*Mean*	*S.D.*	*Mean*	*S.D.*	*Mean*	*S.D.*	*p for trend*	*Mean*	*S.D.*	*Mean*	*S.D.*	*Mean*	*S.D.*	*Mean*	*S.D.*	*p for trend*
**Men (N)**	***894***		***1059***		***1224***		***1509***			***1696***		***1125***		***908***		***957***			***947***		***1015***		***1185***		***1539***		
Age at survey (yrs)	50.0	16.4	55.9	15.9	60.3	14.9	63.3	13.4	**<0.001**	56.4	15.9	57.7	16.1	59.8	15.7	60.9	14.6	**<0.001**	66.7	13.6	61.1	15.0	57.3	15.1	52.1	15.2	**<0.001**
Height (cm)	167.5	6.5	166.7	7.0	166.0	6.7	165.3	6.8	**<0.001**	166.3	7.1	166.2	6.8	166.1	6.7	166.1	6.3	0.900	163.7	6.7	165.6	6.8	166.6	6.7	167.9	6.5	**<0.001**
Body weight (kg)	66.8	11.5	65.5	10.5	65.2	10.4	65.7	10.1	**0.006**	66.1	11.1	65.5	10.5	65.5	9.8	65.6	10.3	0.370	63.3	10.0	65.2	10.3	65.8	10.6	67.4	10.7	**<0.001**
Waist circumference (cm)	85.5	9.4	85.2	9.2	85.7	8.7	86.7	8.2	**<0.001**	85.9	8.9	85.5	9.0	86.1	8.6	86.0	8.7	0.479	85.9	8.7	86.1	8.7	85.5	8.9	85.9	8.9	0.457
BMI	23.8	3.5	23.5	3.3	23.6	3.1	24.0	3.0	**0.004**	23.8	3.3	23.7	3.2	23.7	3.1	23.7	3.2	0.483	23.6	3.1	23.8	3.1	23.7	3.3	23.9	3.3	0.120
Steps per day	6958	4514	7092	4328	7065	5208	6954	4082	0.834	7051	4915	6994	4264	6919	4227	7061	4428	0.890	6276	4262	6839	5236	7193	4236	7448	4360	**<0.001**
Systolic blood pressure	132.6	18.0	133.6	17.6	136.6	17.5	136.9	17.1	**<0.001**	135.9	18.1	134.9	17.3	134.4	17.2	135.3	17.5	0.171	137.2	16.8	136.0	17.6	135.1	17.7	133.7	17.8	**<0.001**
Diastolic blood pressure	82.5	11.5	81.9	11.4	82.0	10.8	81.6	10.7	0.324	82.5	11.3	81.8	10.9	81.4	11.2	81.8	10.5	0.098	81.0	10.8	81.4	10.7	82.2	11.0	82.8	11.4	**0.000**
Serum total cholesterol	196.4	34.4	197.5	33.3	194.3	34.5	194.7	33.6	0.083	194.9	34.2	195.3	33.7	195.8	33.4	196.8	34.2	0.576	188.1	33.4	194.3	33.7	196.2	33.6	200.5	33.8	**<0.001**
Serum HDLcholesterol	54.4	14.2	55.0	15.1	54.9	14.4	54.7	15.7	0.819	54.9	15.4	55.1	14.7	54.4	15.0	54.5	14.2	0.627	53.2	14.4	53.8	14.1	55.0	15.1	56.2	15.5	**<0.001**
Serum LDL cholesterol	117.5	31.4	116.6	30.3	113.5	31.2	112.8	29.6	**<0.001**	113.2	31.1	114.5	30.5	115.3	30.3	117.2	30.0	**0.011**	109.2	30.0	114.7	30.1	114.9	30.0	118.0	31.3	**<0.001**
HbA1C	5.7	0.8	5.7	0.8	5.8	0.8	5.8	0.7	**<0.001**	5.8	0.8	5.8	0.8	5.8	0.7	5.8	0.9	0.107	5.9	0.8	5.9	0.8	5.8	0.8	5.7	0.8	**<0.001**
Anti-hypertensive medication (%)	21.3		26.7		33.2		35.4		**<0.001**	28.0		30.3		32.7		31.3		**0.011**	42.1		33.4		28.0		22.3		**<0.001**
Anti-diabetic medication (%)	5.6		8.4		9.3		9.5		**0.001**	6.9		8.5		9.1		10.4		**0.001**	11.9		10.2		7.8		5.7		**<0.001**
Anti-lipid medication (%)	8.4		11.0		12.9		14.9		**<0.001**	9.2		11.7		14.6		16.0		**<0.001**	14.8		15.0		11.9		9.2		**<0.001**
Current smoking (%)	42.2		32.8		28.3		21.5		**<0.001**	34.2		29.7		25.3		26.2		**<0.001**	21.8		25.1		32.7		35.5		**<0.001**
Past smoking (%)	30.3		37.8		43.1		48.6		**<0.001**	37.4		40.9		45.2		44.5		**<0.001**	48.5		43.0		40.6		36.1		**<0.001**
Current drinking (%)	37.1		33.8		36.6		34.8		0.271	43.2		36.5		32.5		23.4		**<0.001**	24.7		31.6		37.8		42.9		**<0.001**
**Women (N)**	***1589***		***1715***		***1747***		***1628***			***999***		***1715***		***1932***		***2033***			***2116***		***1923***		***1595***		***1045***		
Age at survey (yrs)	49.2	15.3	56.4	15.8	59.9	14.1	64.1	11.9	**<0.001**	57.2	16.3	56.6	16.0	57.6	15.4	58.2	14.2	**0.011**	63.0	14.2	57.6	14.9	53.9	15.2	51.5	14.7	**<0.001**
Height (cm)	154.9	6.3	153.7	6.7	153.3	6.7	152.1	6.2	**<0.001**	152.8	7.1	153.2	6.8	153.5	6.5	154.0	6.1	**<0.001**	151.7	6.7	153.6	6.5	154.5	6.3	155.2	6.0	**<0.001**
Body weight (kg)	53.7	8.9	53.2	8.8	53.1	8.8	53.1	8.3	0.102	53.8	9.6	53.4	8.6	53.3	8.7	52.9	8.2	0.091	52.2	8.5	53.4	8.4	53.9	8.9	54.3	9.0	**<0.001**
Waist circumference (cm)	80.5	10.3	81.3	10.2	81.8	9.8	82.8	9.7	**<0.001**	82.1	10.6	81.9	10.1	81.7	10.1	81.0	9.7	**0.013**	82.0	10.0	81.7	9.9	81.3	10.4	81.0	10.1	**0.026**
BMI	22.4	3.7	22.6	3.6	22.6	3.5	23.0	3.4	**<0.001**	23.1	4.0	22.8	3.5	22.6	3.6	22.3	3.4	**<0.001**	22.7	3.5	22.6	3.5	22.6	3.7	22.6	3.6	0.837
Steps per day	6450	3696	6101	3383	6366	3609	6465	3958	**0.014**	6096	4125	6242	3663	6233	3476	6651	3582	**<0.001**	5824	3706	6398	3559	6637	3588	6837	3766	**<0.001**
Systolic blood pressure	122.8	18.7	127.8	19.1	130.1	18.7	133.3	18.4	**<0.001**	130.5	19.9	128.6	19.1	127.8	18.8	128.3	19.0	**0.003**	131.6	19.1	128.7	19.1	126.1	19.1	125.8	18.1	**<0.001**
Diastolic blood pressure	75.3	10.9	77.1	10.7	78.0	10.7	78.3	10.3	**<0.001**	77.6	10.7	77.0	10.7	76.8	10.5	77.4	10.9	0.174	77.7	10.7	77.2	10.8	76.6	10.7	77.1	10.6	**0.026**
Serum total cholesterol	200.3	34.9	203.5	34.5	207.2	34.3	206.9	34.0	**<0.001**	198.7	34.5	200.6	33.2	205.8	35.1	209.5	34.3	**<0.001**	204.3	34.7	206.1	33.8	204.1	34.6	203.0	35.2	0.096
Serum HDLcholesterol	64.4	15.0	63.8	14.9	64.1	15.9	62.8	15.2	**0.018**	62.0	15.4	62.5	15.1	64.2	15.5	65.4	14.9	**<0.001**	62.2	15.0	63.6	15.3	64.7	15.2	66.0	15.7	**<0.001**
Serum LDL cholesterol	116.2	31.1	118.2	30.7	119.7	30.0	119.5	30.2	**0.004**	114.0	30.0	115.8	29.4	119.6	31.0	121.7	30.9	**<0.001**	118.5	30.8	119.6	30.0	118.3	30.9	116.4	30.4	0.064
HbA1C	5.6	0.6	5.7	0.7	5.7	0.6	5.8	0.7	**<0.001**	5.7	0.7	5.7	0.6	5.7	0.6	5.7	0.6	0.529	5.8	0.6	5.7	0.6	5.6	0.6	5.6	0.7	**<0.001**
Anti-hypertensive medication (%)	15.7		26.3		26.4		32.7		**<0.001**	30.2		27.4		24.4		22.2		**<0.001**	33.9		25.4		19.5		16.9		**<0.001**
Anti-diabetic medication (%)	3.2		4.0		4.2		5.3		**0.002**	4.1		4.4		3.8		4.3		0.496	5.1		5.0		2.8		2.8		**<0.001**
Anti-lipid medication (%)	11.1		16.3		18.5		22.4		**<0.001**	15.1		17.1		16.9		18.3		**0.021**	21.2		18.5		13.7		11.6		**<0.001**
Current smoking (%)	12.4		7.0		5.3		3.1		**<0.001**	7.0		7.3		6.4		6.9		0.351	4.9		5.6		8.2		11.3		**<0.001**
Past smoking (%)	11.4		8.2		6.3		4.4		**<0.001**	7.5		7.1		7.6		7.9		0.229	4.8		7.5		9.3		10.5		**<0.001**
Current drinking (%)	9.1		6.6		5.4		3.9		**<0.001**	8.6		8.2		5.7		3.8		**<0.001**	2.6		5.9		7.7		11.9		**<0.001**

For continuous variables, the general linear model was applied

For categorical variables, the Cochrane-Armitage trend test was applied

Significant *P* values <0.05 were shown in bold characters

### Energy and nutrients intake across the quartiles of food intake patterns

We explored the relationship between nutrients intake and the food intake pattern scores by generalized linear model analysis (Table 4). For all three food intake pattern scores, higher scores were associated with higher total energy intake in both men and women. A higher traditional Japanese pattern score was significantly associated with higher intakes of protein, ω-3, ω-6 fatty acids, dietary fiber, vitamin A, D, B_1_, B_2_, B_6_, B_12_, C, folate, sodium, potassium, calcium and iron in both men and women. Higher Westernized pattern score was associated with higher intake of protein, fat, ω-6 fatty acids, dietary fiber, vitamin A, B_1_, B_2_, B_6_, C, folate, sodium, potassium, calcium, and lower intake of ω-3 fatty acids, carbohydrates, vitamin D and vitamin B_12_. Higher meat and fat pattern score was associated with higher intakes of protein, fat, ω-6 fatty acids, dietary fiber, vitamin B_1_, B_2_, B_6_, sodium, iron and lower intakes of ω-3 fatty acids, vitamin B_12_, C, folate, potassium and calcium in both men and women.

**Table 4:** Energy and nutrient intakes by gender, according to quartiles of the three food intake pattern scores

	Traditional Japanese									Westernized									Meat and fat								
Men	***Q1***		***Q2***		***Q3***		***Q4***			***Q1***		***Q2***		***Q3***		***Q4***			***Q1***		***Q2***		***Q3***		***Q4***		
N	***894***		***1059***		***1224***		***1509***			***1696***		***1125***		***908***		***957***			***947***		***1015***		***1185***		***1539***		
/day	*Mean*	*S.D.*	*Mean*	*S.D.*	*Mean*	*S.D.*	*Mean*	*S.D.*	*p for trend*	*Mean*	*S.D.*	*Mean*	*S.D.*	*Mean*	*S.D.*	*Mean*	*S.D.*	*p for trend*	*Mean*	*S.D.*	*Mean*	*S.D.*	*Mean*	*S.D.*	*Mean*	*S.D.*	*p for trend*
Energy (kcal)	1954	411	2039	409	2117	403	2333	381	<0.001	2189	425	2080	427	2082	414	2166	416	<0.001	2000	393	2042	400	2109	401	2308	422	<0.001
Protein (g)	62.2	16.8	70.5	16.5	76.9	18.0	89.6	20.1	<0.001	77.6	22.1	75.3	20.8	75.3	19.8	78.2	18.9	0.001	73.4	21.5	73.2	19.6	75.3	20.3	82.2	20.3	<0.001
Fat (%energy)	24.9	7.6	23.8	6.9	23.2	6.8	22.6	6.6	<0.001	21.9	6.8	23.3	6.9	24.3	6.8	25.7	6.6	<0.001	19.6	6.3	22.0	6.3	23.9	6.2	26.5	6.9	<0.001
ω-3 fatty acids (g)	1.8	1.1	2.2	1.3	2.6	1.6	3.1	1.7	<0.001	2.7	1.7	2.5	1.6	2.3	1.5	2.3	1.4	<0.001	2.7	1.8	2.5	1.6	2.3	1.5	2.5	1.4	<0.001
ω-6 fatty acids (g)	9.2	4.3	9.5	4.2	9.8	4.3	10.9	4.7	<0.001	10.0	4.5	9.7	4.4	9.8	4.2	10.4	4.7	0.003	7.6	3.8	8.6	3.4	9.8	3.8	12.3	4.8	<0.001
Carbohydrates (%energy)	62.3	8.5	62.2	8.0	62.1	7.9	62.0	7.6	0.782	63.9	8.0	62.2	7.9	61.2	7.7	59.8	7.3	<0.001	65.7	7.8	63.6	7.4	61.8	7.2	59.2	7.8	<0.001
Dietary fiber (g)	10.5	3.7	13.3	4.1	15.8	4.6	21.7	7.0	<0.001	14.5	5.9	15.8	6.5	16.9	6.7	18.6	7.6	<0.001	16.7	7.6	15.8	6.3	16.0	6.7	16.0	6.5	0.019
Vitamin A (μgRAE)	386	520	485	638	540	633	730	832	<0.001	502	708	582	705	575	682	621	682	0.000	513	425	549	557	594	800	569	821	0.055
Vitamin D (μg)	4.8	6.6	7.0	7.8	9.2	9.0	12.1	11.1	<0.001	9.7	10.3	8.8	9.6	8.4	9.2	7.5	7.9	<0.001	11.5	10.9	9.8	10.1	8.3	9.1	6.8	7.8	<0.001
Vitamin B_1_ (mg)	0.9	1.0	0.9	0.7	1.0	0.6	1.1	0.7	<0.001	0.9	0.8	1.0	0.7	1.0	0.6	1.1	0.8	<0.001	0.9	0.7	1.0	0.8	1.0	0.8	1.1	0.7	<0.001
VitaminB_2_ (mg)	1.1	0.9	1.2	0.8	1.3	0.6	1.6	0.7	<0.001	1.2	0.8	1.3	0.7	1.3	0.6	1.5	0.8	<0.001	1.4	0.7	1.3	0.8	1.3	0.8	1.3	0.8	0.165
Vitamin B_6_ (mg)	1.0	0.9	1.2	1.0	1.4	0.7	1.8	1.1	<0.001	1.4	1.0	1.4	0.9	1.3	0.9	1.5	1.0	0.071	1.4	0.9	1.4	0.9	1.3	0.8	1.5	1.2	0.011
Vitamin B_12_ (μg)	4.6	5.3	6.4	7.9	7.6	7.3	9.8	8.9	<0.001	8.1	8.7	7.4	7.3	7.1	7.7	6.8	7.2	0.000	9.3	8.5	7.9	7.5	6.9	7.1	6.5	8.2	<0.001
Folate (μg)	208	91	275	138	325	117	438	165	<0.001	317	176	322	149	334	152	348	148	<0.001	350	171	325	140	320	144	322	176	<0.001
VitaminC (mg)	58.9	56.7	82.9	56.4	106.0	63.5	154.6	99.4	<0.001	91.2	63.6	102.5	74.1	116.2	83.0	133.7	110.6	<0.001	130.0	102.6	110.0	77.8	103.1	79.5	95.2	71.4	<0.001
Sodium (mg)	3482	1319	4021	1304	4553	1377	5568	1693	<0.001	4579	1677	4496	1687	4467	1560	4668	1674	0.032	4257	1618	4389	1602	4471	1535	4914	1746	<0.001
Potassium (mg)	1689	519	2124	495	2494	543	3303	837	<0.001	2338	810	2460	830	2615	912	2811	934	<0.001	2639	1012	2465	846	2476	859	2509	822	<0.001
Calcium (mg)	363	191	460	217	535	213	664	248	<0.001	450	213	512	229	571	249	638	273	<0.001	574	275	538	242	516	249	499	227	<0.001
Iron (mg)	5.9	1.9	7.4	2.1	8.5	2.1	10.9	3.0	<0.001	8.5	3.0	8.5	3.0	8.5	3.1	8.7	3.0	0.511	8.7	3.1	8.3	3.1	8.4	3.0	8.7	2.9	0.004
**Women**																											
N	***1589***		***1715***		***1747***		***1628***			***999***		***1715***		***1932***		***2033***			***2116***		***1923***		***1595***		***1045***		
Energy (kcal)	1542	320	1640	316	1744	309	1905	298	<0.001	1724	342	1661	339	1685	340	1764	325	<0.001	1637	335	1673	325	1738	328	1875	319	<0.001
Protein (g)	52.7	13.8	60.3	14.4	66.8	15.0	76.8	16.2	<0.001	63.7	17.3	63.1	18.1	63.8	17.0	65.9	16.6	<0.001	62.2	17.8	62.5	16.5	65.2	16.6	70.0	17.1	<0.001
Fat (%energy)	27.1	7.5	25.8	7.2	25.1	6.9	24.3	6.6	<0.001	23.1	6.9	24.5	7.1	26.1	7.1	27.2	6.9	<0.001	22.2	6.6	25.2	6.4	27.6	6.6	29.7	6.9	<0.001
ω-3 fatty acids (g)	1.5	1.0	1.9	1.2	2.2	1.3	2.6	1.4	<0.001	2.3	1.3	2.1	1.3	2.1	1.3	2.0	1.3	<0.001	2.2	1.4	2.0	1.3	2.0	1.2	2.1	1.2	<0.001
ω-6 fatty acids (g)	7.7	3.5	8.0	3.6	8.8	3.9	9.7	4.2	<0.001	8.7	3.9	8.2	3.8	8.5	4.0	8.8	3.9	<0.001	7.1	3.5	8.1	3.4	9.3	3.7	11.3	4.2	<0.001
Carbohydrates (%energy)	59.2	8.4	59.4	8.1	59.4	7.9	59.5	7.6	0.706	62.1	8.1	60.3	8.2	58.8	7.9	57.9	7.5	<0.001	62.6	7.8	59.8	7.4	57.3	7.5	55.3	7.7	<0.001
Dietary fiber (g)	10.3	3.3	13.4	3.9	16.4	4.3	21.7	6.2	<0.001	13.3	5.2	14.8	6.1	15.6	6.1	17.1	6.2	<0.001	15.7	6.4	15.2	6.0	15.3	5.9	15.7	6.3	0.025
Vitamin A (μgRAE)	362	335	472	396	586	686	714	596	<0.001	471	773	521	533	537	430	575	501	<0.001	513	417	543	528	548	579	542	705	0.165
Vitamin D (μg)	4.5	6.2	6.8	7.7	8.2	8.1	10.6	9.4	<0.001	7.8	8.3	8.0	8.7	7.6	8.2	7.0	7.8	0.001	9.0	8.9	7.5	8.1	7.0	8.0	5.7	6.7	<0.001
Vitamin B_1_ (mg)	0.7	0.5	0.8	0.5	0.9	0.5	1.0	0.5	<0.001	0.7	0.4	0.8	0.6	0.8	0.5	0.9	0.5	<0.001	0.8	0.6	0.8	0.4	0.9	0.5	1.0	0.6	<0.001
VitaminB_2_ (mg)	0.9	0.5	1.1	0.6	1.2	0.5	1.4	0.5	<0.001	1.0	0.5	1.1	0.6	1.2	0.6	1.2	0.6	<0.001	1.2	0.6	1.1	0.5	1.1	0.5	1.2	0.6	<0.001
Vitamin B_6_ (mg)	0.8	0.6	1.1	0.7	1.2	0.6	1.5	0.6	<0.001	1.1	0.7	1.2	0.7	1.2	0.7	1.2	0.6	0.012	1.2	0.7	1.1	0.7	1.1	0.5	1.2	0.7	<0.001
Vitamin B_12_ (μg)	3.8	4.3	5.5	5.7	6.4	6.2	7.9	6.5	<0.001	6.5	7.1	6.2	6.2	5.7	5.5	5.6	5.5	<0.001	7.1	6.7	5.7	5.7	5.3	5.2	4.9	5.5	<0.001
Folate (μg)	200	70	273	89	331	109	431	130	<0.001	288	129	302	134	311	128	324	133	<0.001	327	136	302	126	301	129	299	134	<0.001
VitaminC (mg)	64.3	50.0	95.9	58.6	121.9	69.2	167.6	90.3	<0.001	89.6	58.2	105.6	73.1	113.2	77.4	129.5	87.1	<0.001	131.6	89.5	109.2	74.1	100.4	66.1	99.4	69.9	<0.001
Sodium (mg)	2949	1016	3512	1072	4076	1144	4978	1413	<0.001	3967	1420	3824	1389	3816	1372	3955	1367	0.001	3653	1327	3810	1372	3957	1348	4372	1441	<0.001
Potassium (mg)	1596	450	2080	487	2493	531	3204	756	<0.001	2054	687	2241	794	2375	811	2554	826	<0.001	2425	854	2300	797	2300	769	2349	805	<0.001
Calcium (mg)	359	177	462	203	556	212	672	238	<0.001	401	180	466	230	529	232	594	245	<0.001	552	257	507	229	495	230	473	215	<0.001
Iron (mg)	5.4	1.5	6.9	2.1	8.2	2.0	10.2	2.7	<0.001	7.5	2.5	7.7	2.9	7.7	2.7	7.8	2.7	0.115	7.8	3.0	7.5	2.6	7.7	2.7	7.9	2.6	0.001

For continuous variables, the general linear model was applied

### Association of each food intake pattern with waist circumference, BMI, blood pressure and blood lipid profiles

Table 5 shows mean waist circumference, BMI, blood pressure, HbA1c and blood lipid profiles according to the quartiles for each food intake pattern. The multivariable adjusted geometric means for waist circumference (*P* for trend = 0.008) and BMI (*P* for trend = 0.001) in men, and mean DBP (*P* for trend = 0.019) in women were significantly increased according to the lowest to the highest quartile of traditional Japanese pattern. Men in the highest quartile of Westernized pattern had lower SBP (P for trend = 0.003), higher TC (*P* for trend = 0.047) and higher LDL-C (*P* for trend = 0.006), while women in the highest quartile of Westernized pattern had lower waist circumference (P for trend = 0.039), lower BMI (*P* for trend <0.001), lower SBP (*P* for trend = 0.001) and higher TC, HDL-C and LDL-C (*P* for trend <0.001 for all). Compared with the lowest quartile of the meat and fat pattern, those in the highest quartile had higher waist circumference (P for trend = 0.016), SBP (*P* for trend = 0.013), DBP (*P* for trend = 0.003), TC (P for trend <0.001) and LDL-C (*P* for trend <0.001) in men and higher waist circumference (P for trend <0.001), BMI (*P* for trend <0.001), TC (*P* for trend = 0.001) and LDL-C (*P* for trend = 0.013) in women. No association between HbA1c and food intake patterns was observed in both men and women.

**Table 5:** Subject characteristics adjusted for age, medication status, smoking status, drinking, and steps count, according to quartiles of the three food intake pattern scores

	Traditional Japanese								Westernized										Meat and fat								
	***Q1***		***Q2***		***Q3***		***Q4***			***Q1***		***Q2***		***Q3***		***Q4***			***Q1***		***Q2***		***Q3***		***Q4***		
	*Mean*	*S.E.*	*Mean*	*S.E.*	*Mean*	*S.E.*	*Mean*	*S.E.*	*p for trend*	*Mean*	*S.E.*	*Mean*	*S.E.*	*Mean*	*S.E.*	*Mean*	*S.E.*	*p for trend*	*Mean*	*S.D.*	*Mean*	*S.D.*	*Mean*	*S.D.*	*Mean*	*S.D.*	*p for trend*
**Men (N)**	***895***		***1061***		***1228***		***1509***			***1698***		***1127***		***909***		***959***			***947***		***1015***		***1185***		***1539***		
Waist circumference (cm)	85.8	0.3	85.3	0.3	85.6	0.2	86.4	0.2	**0.008**	86.0	0.2	85.5	0.3	85.9	0.3	85.9	0.3	0.516	85.2	0.3	85.9	0.3	85.6	0.2	86.4	0.2	**0.016**
BMI (kg/m^2^)	23.7	0.1	23.5	0.1	23.7	0.1	24.0	0.1	**0.001**	23.9	0.1	23.7	0.1	23.7	0.1	23.7	0.1	0.333	23.6	0.1	23.7	0.1	23.7	0.1	23.9	0.1	0.081
Systolic blood pressure	136.1	0.5	134.7	0.5	135.6	0.5	134.8	0.4	0.182	136.3	0.4	135.0	0.5	133.9	0.5	134.9	0.5	**0.003**	133.8	0.5	135.1	0.5	135.4	0.5	136.1	0.4	**0.013**
Diastolic blood pressure	82.7	0.4	82.1	0.3	81.9	0.3	81.5	0.3	0.128	82.2	0.3	81.8	0.3	81.5	0.4	82.2	0.4	0.338	81.0	0.4	81.5	0.3	82.1	0.3	82.7	0.3	**0.003**
Serum total cholesterol	195.1	1.1	197.2	1.0	194.7	1.0	195.4	0.9	0.305	194.1	0.8	195.2	1.0	196.4	1.1	197.8	1.1	**0.047**	189.5	1.1	194.8	1.0	195.9	1.0	199.6	0.9	**<0.001**
Serum HDLcholesterol	54.3	0.5	55.1	0.4	54.8	0.4	54.7	0.4	0.691	54.3	0.3	55.0	0.4	54.6	0.5	55.5	0.5	0.170	54.5	0.5	54.1	0.4	54.8	0.4	55.4	0.4	0.199
Serum LDL cholesterol	115.9	1.0	115.9	0.9	114.0	0.9	113.8	0.8	0.191	113.0	0.7	114.5	0.9	115.6	1.0	117.1	1.0	**0.006**	110.0	1.0	115.1	0.9	114.7	0.9	117.4	0.8	**<0.001**
HbA1C	5.78	0.02	5.77	0.02	5.79	0.02	5.78	0.02	0.902	5.81	0.02	5.77	0.02	5.75	0.02	5.77	0.02	0.158	5.74	0.02	5.79	0.02	5.77	0.02	5.80	0.02	0.125
**Women (N)**	***1589***		***1716***		***1748***		***1628***			***999***		***1715***		***1932***		***2033***			***2116***		***1923***		***1595***		***1045***		
Waist circumference (cm)	82.0	0.2	81.3	0.2	81.4	0.2	81.7	0.2	0.170	81.9	0.3	81.8	0.2	81.7	0.2	81.1	0.2	**0.039**	80.7	0.2	81.7	0.2	82.1	0.2	82.4	0.3	**<0.001**
BMI (kg/m^2^)	22.7	0.1	22.5	0.1	22.5	0.1	22.7	0.1	0.138	23.0	0.1	22.7	0.1	22.6	0.1	22.4	0.1	**<0.001**	22.4	0.1	22.6	0.1	22.8	0.1	22.9	0.1	**<0.001**
Systolic blood pressure	128.1	0.4	128.4	0.4	128.7	0.4	129.1	0.4	0.321	130.3	0.5	128.9	0.4	127.8	0.4	128.1	0.4	**0.001**	127.9	0.4	128.6	0.4	128.6	0.4	129.6	0.5	0.062
Diastolic blood pressure	76.5	0.3	77.2	0.3	77.6	0.2	77.3	0.3	**0.019**	77.5	0.3	77.1	0.3	76.8	0.2	77.4	0.2	0.229	76.9	0.2	77.2	0.2	77.1	0.3	77.9	0.3	0.115
Serum total cholesterol	204.1	0.9	204.4	0.8	205.8	0.8	203.7	0.8	0.287	199.3	1.0	201.4	0.8	205.6	0.7	208.7	0.7	**<0.001**	202.1	0.7	206.1	0.8	205.3	0.8	205.3	1.0	**0.001**
Serum HDLcholesterol	63.5	0.4	63.8	0.4	64.3	0.4	63.5	0.4	0.338	62.0	0.5	62.3	0.4	64.3	0.3	65.4	0.3	**<0.001**	63.3	0.3	63.5	0.3	64.0	0.4	64.8	0.5	0.070
Serum LDL cholesterol	118.8	0.8	118.7	0.7	118.7	0.7	117.5	0.7	0.531	114.5	0.9	116.6	0.7	119.4	0.7	121.0	0.7	**<0.001**	116.8	0.7	119.7	0.7	119.1	0.7	118.3	0.9	**0.013**
HbA1C	5.68	0.01	5.70	0.01	5.69	0.01	5.72	0.01	0.173	5.73	0.02	5.69	0.01	5.69	0.01	5.69	0.01	0.295	5.68	0.01	5.70	0.01	5.70	0.01	5.73	0.02	0.063

The general linear model adjusted for participant age, steps count, medication status (hypertension, diabetes, and dyslipidemia), smoking and drinking status was applied

Significant *P* values <0.05 were shown in bold characters

### Association of each food intake pattern with hypertension, diabetes, hypercholesterolemia and elevated LDL cholesterol

Table 6 showed the logistic regression analysis for the association of each food intake pattern with cardiovascular disease risk. Traditional Japanese pattern was related to a lower prevalence of hypertension in men and not in women. The multivariate-adjusted ORs (95% CI) for the lowest through highest quartiles of the traditional Japanese pattern were 1.00 (reference), 0.75 (0.59–0.95), 0.83 (0.65–1.04) and 0.67 (0.53–0.84), respectively (trend *P* = 0.003). Westernized pattern was associated with higher prevalence of hypercholesterolemia and elevated LDL cholesterol in women, but not in men. The multivariate-adjusted ORs (95% CI) comparing the highest quartile to the lowest were Q2: 1.13 (0.87–1.46), Q3: 1.33 (1.04–1.71) and Q4: 1.80 (1.41–2.29) for hypercholesterolemia (trend *P* < 0.001), and Q2: 1.19 (0.86–1.62), Q3: 1.43 (1.06–1.93) and Q4: 1.75 (1.30–2.35) for elevated LDL cholesterol (trend P < 0.001). Also, higher meat-fat pattern score was positively associated with hypertension, diabetes and hypercholesterolemia in men, but not in women (Table 6).

**Table 6:** Association of each food intake pattern with hypertension, diabetes, hypercholesterolemia and elevated LDL cholesterol

	Traditional Japanese					Westernized					Meat and fat				
	***Q1***	***Q2***	***Q3***	***Q4***	*p for trend*	***Q1***	***Q2***	***Q3***	***Q4***	*p for trend*	***Q1***	***Q2***	***Q3***	***Q4***	*p for trend*
**Men (N)**	***895***	***1061***	***1228***	***1509***		***1698***	***1127***	***909***	***959***		***947***	***1015***	***1185***	***1539***	
Hypertension															
Prevalence: n (%)	445 (49.8)	546 (51.6)	738 (60.3)	918 (60.8)		959 (56.5)	633 (56.3)	517 (56.9)	538 (56.2)		611 (64.5)	596 (58.7)	650 (54.9)	790 (51.3)	
OR^a^ (95% CI)	1 (ref)	0.75 (0.59–0.95)	0.83 (0.65–1.04)	0.67 (0.53–0.84)	**0.003**	1 (ref)	0.93 (0.76–1.14)	0.86 (0.69–1.06)	0.84 (0.68–1.05)	0.169	1 (ref)	1.13 (0.88–1.45)	1.20 (0.94–1.53)	1.41 (1.11–1.79)	**0.008**
Diabetes															
Prevalence: n (%)	110 (12.3)	148 (14.0)	214 (17.5)	271 (18.0)		245 (14.4)	176 (15.6)	149 (16.4)	173 (18.1)		179 (18.9)	199 (19.6)	184 (15.5)	181 (11.8)	
OR^a^ (95% CI)	1 (ref)	0.69 (0.48–1.00)	0.90 (0.64–1.27)	0.80 (0.57–1.11)	0.300	1 (ref)	0.92 (0.69–1.23)	0.87 (0.64–1.19)	0.92 (0.68–1.25)	0.839	1 (ref)	1.62 (1.17–2.25)	1.52 (1.09–2.12)	1.38 (0.98–1.95)	**0.021**
Hypercholesterolemia															
Prevalence: n (%)	170 (19.0)	219 (20.7)	266 (21.7)	355 (23.5)		315 (18.6)	231 (20.5)	220 (24.2)	244 (25.5)		197 (20.8)	243 (23.9)	250 (21.1)	320 (20.8)	
OR^a^ (95% CI)	1 (ref)	1.01 (0.75–1.38)	0.94 (0.69–1.27)	0.94 (0.70–1.27)	0.853	1 (ref)	1.04 (0.79–1.36)	1.20 (0.90–1.60)	1.22 (0.92–1.62)	0.415	1 (ref)	1.45 (1.02–2.07)	1.34 (0.94–1.89)	1.64 (1.17–2.29)	**0.036**
Elevated LDL cholesterol															
Prevalence: n (%)	161 (18.0)	200 (18.9)	234 (19.1)	314 (20.8)		270 (15.9)	213 (18.9)	206 (22.7)	220 (23.0)		190 (20.1)	217 (21.4)	223 (18.8)	279 (18.1)	
OR^a^ (95% CI)	1 (ref)	0.94 (0.67–1.30)	0.78 (0.56–1.10)	0.78 (0.56–1.09)	0.281	1 (ref)	1.19 (0.88–1.62)	1.40 (1.02–1.92)	1.18 (0.85–1.64)	0.228	1 (ref)	1.11 (0.75–1.64)	1.07 (0.73–1.57)	1.28 (0.89–1.85)	0.462
**Women (N)**	***1589***	***1716***	***1748***	***1628***		***999***	***1715***	***1932***	***2033***		***2116***	***1923***	***1595***	***1045***	
Hypertension															
Prevalence: n (%)	439 (27.6)	697 (40.6)	778 (44.5)	835 (51.3)		461 (46.1)	727 (42.4)	782 (40.5)	779 (38.3)		1073 (50.7)	798 (41.5)	547 (34.3)	331 (31.7)	
OR^a^ (95% CI)	1 (ref)	1.05 (0.83–1.32)	1.13 (0.91–1.42)	1.03 (0.82–1.30)	0.507	1 (ref)	0.96 (0.75–1.23)	0.90 (0.70–1.14)	0.88 (0.69–1.11)	0.142	1 (ref)	1.05 (0.86–1.27)	1.00 (0.81–1.22)	1.03 (0.81–1.31)	0.840
Diabetes															
Prevalence: n (%)	100 (6.3)	141 (8.2)	141 (8.1)	171 (10.5)		97 (9.7)	146 (8.5)	146 (7.6)	164 (8.1)		207 (9.8)	177 (9.2)	102 (6.4)	67 (6.4)	
OR^a^ (95% CI)	1 (ref)	1.02 (0.70–1.48)	0.86 (0.59–1.26)	0.95 (0.66–1.38)	0.521	1 (ref)	0.78 (0.54–1.13)	0.71 (0.49–1.02)	0.75 (0.52–1.08)	0.061	1 (ref)	1.14 (0.85–1.55)	1.09 (0.77–1.53)	1.31 (0.88–1.96)	0.231
Hypercholesterolemia															
Prevalence: n (%)	375 (23.6)	497 (29.0)	588 (33.7)	585 (35.9)		255 (25.5)	482 (28.1)	586 (30.3)	722 (35.5)		721 (34.1)	617 (32.1)	443 (27.8)	264 (25.3)	
OR^a^ (95% CI)	1 (ref)	0.92 (0.74–1.14)	1.02 (0.83–1.26)	0.84 (0.67–1.06)	0.237	1 (ref)	1.13 (0.87–1.46)	1.33 (1.04–1.71)	1.80 (1.41–2.29)	**<0.001**	1 (ref)	1.14 (0.94–1.38)	1.21 (0.99–1.48)	1.21 (0.96–1.53)	0.567
Elevated LDL cholesterol															
Prevalence: n (%)	302 (19.0)	426 (24.8)	491 (28.1)	514 (31.6)		218 (21.8)	418 (24.4)	505 (26.1)	592 (29.1)		631 (29.8)	516 (26.8)	373 (23.4)	213 (20.4)	
OR^a^ (95% CI)	1 (ref)	1.06 (0.82–1.37)	1.12 (0.87–1.45)	1.05 (0.80–1.38)	0.755	1 (ref)	1.19 (0.86–1.62)	1.43 (1.06–1.93)	1.75 (1.30–2.35)	**<0.001**	1 (ref)	0.99 (0.78–1.24)	1.15 (0.91–1.46)	1.06 (0.80–1.40)	0.333

^**a**^ The logistic regression analysis adjusted for participant age, waist circumference, BMI, steps count, medication status (hypertension, diabetes, and dyslipidemia), smoking and drinking status was applied

OR (95% CI): Adjusted odds ratio (95% Confidence Interval)

Significant *P* values <0.05 were shown in bold characters

## Discussion

In this cross-sectional analysis of the Japan NHNS 2012 data, we identified three food intake patterns derived by principal component analysis: the traditional Japanese pattern, Westernized pattern, meat and fat pattern. Food intake patterns showed differences in associations between CVRFs among men and women. The traditional Japanese pattern was significantly associated with increased BMI and waist circumference, but significantly lower prevalence of hypertension in men. This association may be partly explained by higher energy intake, but with higher vegetable as well as potatoes consumption, which are rich in potassium [30] and contributed to lower blood pressure [31]. However, the traditional Japanese pattern for women was found to have a positive association with diastolic blood pressure. High salt intake (such as from miso soup and Japanese pickles intake) in the highest quartile of traditional Japanese pattern in women may have contributed to raised diastolic blood pressure [32, 33].

Westernized pattern was inversely associated with SBP in men, waist circumference in both men and women, and BMI (in women). The Westernized pattern identified in our study is somewhat similar to previously reported ‘bread-dairy pattern’ or ‘Westernized breakfast pattern’, mainly comprising bread and danish, butter and margarine, milk, cheese and yogurt, fresh fruits but low intakes of rice, which was inversely related with abdominal obesity, BMI and blood pressure [34–37]. In our study population, subjects in the highest quartile of the Westernized pattern consumed greater amount of potassium intake than those in the lowest quartile. Because high potassium intake is a known protective factor for hypertension [31], the higher potassium intake from fresh fruits in the high Westernized pattern score group may have contributed to lower blood pressure. Additionally, foods or nutrients contributed to westernized breakfast pattern such as milk and yogurt, which are rich source of calcium, have also been shown to decrease blood pressure [38]. On the other hand, the Westernized pattern was positively associated with HDL-C (in women), TC and LDL-C in both men and women, and positively associated with hypercholesterolemia and elevated LDL cholesterol in women. This may be due to high fat intake among the highest quartile of Westernized pattern (Table 4) [39].

Interestingly, a simultaneous increase in both HDL and LDL cholesterol was observed with the higher Westernized pattern score in women, consistent with the finding reported by JY Shin et al., 2013 [35]. It has been reported that the average HDL-C levels are indeed high among Japanese people in general, due to genetic deficiency of cholesteryl ester transfer protein (CETP) [40]. In spite of high HDL-C levels, we speculate that these certain number of subjects with CETP deficiency had increased risk of dyslipidemia. Furthermore, this direct association of increased HDL cholesterol with the Westernized pattern might be mediated by lower carbohydrates [41] and higher saturated fat intake [42] and butter [43] among those in the highest quartile of the Westernized pattern.

As expected, meat and fat pattern identified in our study was positively associated with waist circumference, BMI, blood pressure, TC and LDL-C in both men and women, and higher prevalence of hypertension, diabetes and hypercholesterolemia in men after additional adjustment for waist circumference and BMI [44–47]. These positive associations may partly be attributable to the unhealthy cardiovascular risk constituents in meat and fat pattern (such as red meat and saturated fats and cholesterol) [48]. Our result is comparable to a cohort study in Japan that reported a pattern with high meat intakes was closely related to an increased risk of cardiovascular diseases [49].

However, the present study has some limitations. First, as the association was derived from the cross-sectional study, the causal relationship between food intake patterns and the cardiovascular risk factors could not be determined, and may have possibility of reverse causality. Awareness of previously diagnosed hypertension or dyslipidemia can alter their food intake patterns, such as an increase in the consumption of vegetables in hypertensive subjects [50]. However, the significant association persisted after adjusting for medication for hypertension, diabetes and hyperlipidemia. Second, as the Japan NHNS is usually conducted in November, the observed food intake patterns may not accurately reflect seasonal variations in food intake and availability. Third, although the food intake was estimated through a self-reported dietary record data and checking of the records were conducted by trained dieticians, under or over reporting of intake is inevitable. Last, because food intake patterns were derived from the one-day dietary record with a diverse variables of food items, explained variation in food groups of the principal component analysis was low compared to previous studies using FFQs. Because FFQs are developed to assess the habitual food intakes of the specific population of research subjects, the possible food intake patterns may have been pre-defined by their habitual intake which give rise to higher explained variance of each pattern compared to one-day dietary record. Previous study of dietary patterns by Hamer M et al. using weighed food record also showed a similar trend of low percentage of explained variance [51]. Moreover, a study of dietary patterns by McCann SE et al. using FFQs, also observed that the explained variance increased as the number of food groups decreased [52]. Despite these limitations, our study was based on a large-scale, nationally representative health and nutrition survey data, which was highly standardized in obtaining socio demographic and lifestyle characteristics as well as biological cardiovascular risk factors.

## Conclusion

In summary, three major food intake patterns identified in this cross-sectional analysis, were found to have significant associations with cardiovascular risk factors. The traditional Japanese pattern showed protective effect against hypertension in men, Westernized pattern was positively associated with dyslipidemia in women, while meat-fat pattern was related to all cardiovascular risk factors in men. The association between cardiovascular disease risk factors and food intake patterns derived from one-day dietary records of the National Health and Nutrition Survey was similar to previous cohort studies examining habitual intake through FFQs.

## Abbreviations

BMI: Body mass index
CETP: Cholesteryl ester transfer protein
CVD: Cardiovascular disease
CVRF: Cardiovascular risk factors
DASH: Dietary approaches to stop hypertension
DBP: Diastolic blood pressure
FFQs: Food frequency questionnaires
HbA1c: Hemoglobin A1c
HDL-C: High density lipoprotein cholesterol
J-NHNS: The Japan National Health and Nutrition Survey
LDL-C: low density lipoprotein cholesterol
NGSP: National Glyco-hemoglobin Standardization Program
OR: Odds ratio
PCA: Principal component analysis
SBP: Systolic blood pressure
TC: Total cholesterol
WC: Waist circumference
